# Anti-Parkinson Drug Biperiden Inhibits Enzyme Acetylcholinesterase

**DOI:** 10.1155/2017/2532764

**Published:** 2017-07-13

**Authors:** Adam Kostelnik, Alexander Cegan, Miroslav Pohanka

**Affiliations:** ^1^Faculty of Chemical Technology, University of Pardubice, Studentska 95, 53210 Pardubice, Czech Republic; ^2^Faculty of Military Health Sciences, University of Defence, Trebesska 1575, 50001 Hradec Kralove, Czech Republic

## Abstract

Biperiden is a drug used in Parkinson disease treatment and it serves also as an antiseizures compound in organophosphates poisoning. It acts as antagonist of muscarinic receptor activated by acetylcholine while the enzyme acetylcholinesterase (AChE) cleaves acetylcholine in synaptic junction into choline and acetic acid. This enzyme is inhibited by various compounds; however there has not been proposed evidence about interaction with biperiden molecule. We investigated this interaction using standard Ellman's assay and experimental findings were critically completed with an in silico prediction by SwissDock docking software. Uncompetitive mechanism of action was revealed from Dixon plot and inhibition constant (*K*_*i*_) was calculated to be 1.11 mmol/l. The lowest predicted binding energy was −7.84 kcal/mol corresponding to H-bond between biperiden molecule and Tyr 341 residuum in protein structure of AChE. This interaction seems to be further stabilized by *π*-*π* interaction with Tyr 72, Trp 286, and Tyr 341. In conclusion, biperiden appears as a very weak inhibitor but it can serve as a lead structure in a pharmacological research.

## 1. Introduction

Termination of neurotransmission in cholinergic nerves or neuromuscular junctions is done by an enzyme AChE. Mechanism of action is in cleaving of acetylcholine, neurotransmitter interacting with acetylcholine muscarinic receptor (mAChR) and nicotinic receptor (nAChR) [1, 2]. AChE is a target for many toxic compounds like organophosphorus and carbamate pesticides (e.g., parathion, malathion, and carbofuran), warfare agents (e.g., sarin, soman, and VX), or some toxins like aflatoxin B1 [3–7]. Big and important group of AChE inhibitors is created by anti-Alzheimer drugs as donepezil, galantamine, rivastigmine, or huperzine A [8–10].

**Figure 1:**
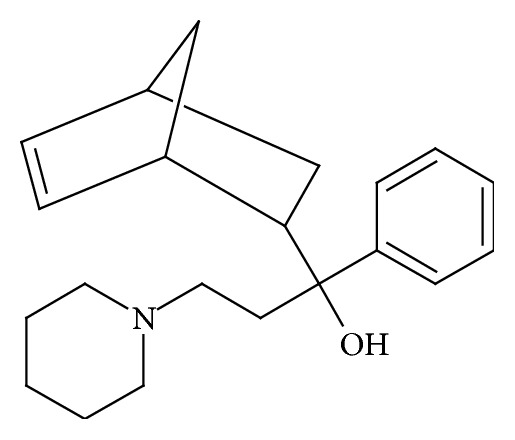
Chemical structure of biperiden molecule.

Biperiden also known under tradename Akineton®, a compound with proper chemical name alpha-bicyclo[2.2.1]hept-5-en-2-yl-alpha-phenyl-1-piperidinepropanol (Figure 1), is an anticholinergic drug used in treatment of Parkinson disease and neuroleptic-induced extrapyramidal motor side effects [11]. It acts as a muscarinic receptor antagonist with high affinity for the M1 muscarinic receptor [12]. Furthermore, it can be used as antiseizures compound in poisoning by organophosphates [13, 14].

Despite the fact that biperiden is used in therapy of parkinsonism, there is no evidence about possible interaction with AChE itself. Some structural motives in the biperiden resemble another AChE inhibitor, compound known as huperzine A. The fact leads us to the idea that biperiden can act as inhibitor and we hypothesize a possible interaction with AChE.

## 2. Material and Methods

### 2.1. Chemicals

Acetylcholinesterase as lyophilized powder (electric eel, activity ≥ 1000 units/mg of protein), acetylthiocholine chloride, 5,5′-dithiobis(2-nitrobenzoic) acid (DTNB), and phosphate buffer saline (PBS) 7.4 were purchased from Sigma-Aldrich (St. Louis, MO, USA). Biperiden lactate (5 mg/ml) in one-milliliter ampules was obtained from Knoll AG (Ludwigshafen, Germany). Deionized water was prepared by Aqua Osmotic device (Tisnov, Czech Republic).

### 2.2. Enzymatic Assay with Biperiden

Ellman's method was chosen for the enzyme activity assay and it was performed as follows: 400 *μ*l of DTNB, 25 *μ*l of AChE, 375 *μ*l of PBS 7.4, 100 *μ*l of PBS 7.4 or biperiden, and 100 *μ*l of acetylcholine chloride were pipetted into standard spectrophotometric cuvette. Absorbance was measured immediately after addition of substrate and then after 2 min of reaction. Enzyme activity was then calculated using extinction coefficient for 5-thio-2-nitrobenzoic acid *ε* = 14,150 l × mol^−1^  ×  cm^−1^ [15]. Concentration of biperiden was calculated to whole volume of reaction medium in cuvette.

### 2.3. Data Processing

Dixon plot was created in Origin software (OriginLab, Northampton, MA, USA). *K*_*i*_ for uncompetitive inhibition was calculated from Dixon plot as follows: Slope = 1/*V*_max_ × *K*_*i*_. *V*_max_ for AChE was obtained experimentally (as described in previous section) and calculated in Origin software (OriginLab, Northampton, MA, USA) using nonlinear Hill fitting with coefficient of cooperativity *n* = 1.

### 2.4. Docking of Biperiden to AChE

SwissDock server (Swiss Institute of Bioinformatics, University of Lausanne, Switzerland) was used for in silico prediction of the lowest free binding energy. The calculation was running online (accessible from http://www.swissdock.ch/) in the Internet browser. Crystal structure of AChE (1C2B) [16] was taken in PDB format and biperiden ligand in ZINC format as required for calculation [17]. UCSF Chimera 1.11.2 software was used for visualization of the results and creating 3D images.

## 3. Results and Discussion

**Figure 2:**
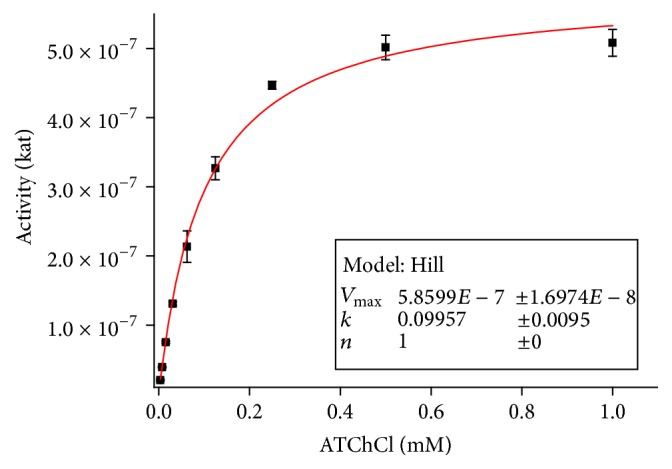
Saturation curve for AChE and acetylthiocholine as a substrate. Error bars indicate standard deviation for *n* = 3.

**Figure 3:**
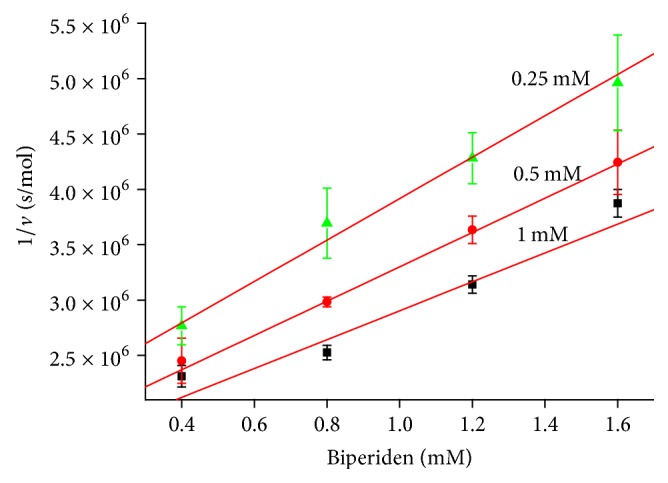
Dixon plot for AChE with different concentrations of substrate (indicated above each line). Error bars indicate standard deviation for *n* = 3.

Biperiden proved to be inhibitor of AChE as seen from the results. From Dixon plot, uncompetitive mechanism of AChE inhibition was revealed (Figure 3). This type of inhibition is very rare and it is more probable for multifold substrate reactions. More typical mechanism for AChE is noncompetitive or competitive inhibition [18]. *K*_*i*_ for biperiden and AChE was calculated to be 1.11 ± 0.20 mmol/l, which equals IC_50_ in this type of inhibition [19].  Figure 2 is displaying saturation curve from which *V*_max_ for *K*_*i*_ calculation was obtained as described above. Data obtained from experiment are summarized in Table 1.

**Figure 4:**
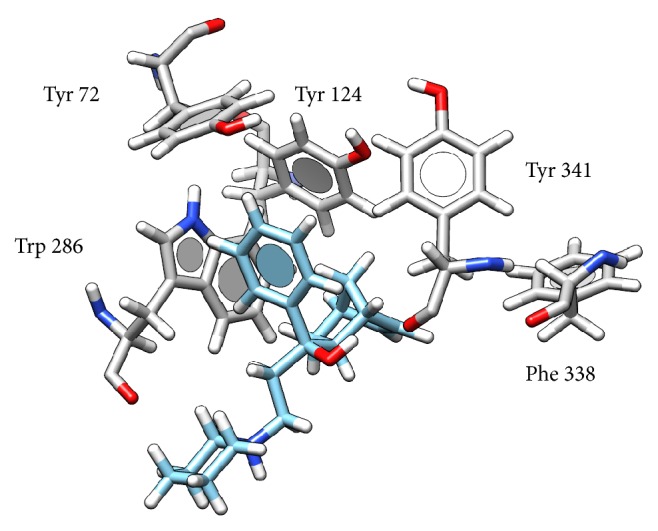
Possible interaction of biperiden with AChE. Atom color: white = H, grey (in AChE) or light blue (in biperiden) = C, blue = N, and red = O.

Interaction of biperiden with AChE was studied using SwissDock server. The lowest binding energy Δ*G* was equal to −7.84 kcal/mol and corresponds to interaction between biperiden and peripheral anionic subsite. In the lowest energy, there is predicted H-bond between hydroxyl group in biperiden molecule and O atom in Tyr 341 (2.24 Å). This seems to be stabilized by *π*-*π* interaction of benzene ring in biperiden with aromatic amino acids of peripheral anionic subsite Tyr 72 (3.43 Å), Trp 286 (3.18 Å), and Tyr 341 (3.05 Å) (Figure 4). As seen from the quoted papers [20, 21], T-shape geometry (or face to age) interaction is the most common between two aromatic systems and it was found to be most abundant interaction in present work. On the other hand, face to face interaction is very rare due to electrostatic repulsion and it was not observed in this study. As bicycloheptenyl contains double bond, it does not seem to provide any further stabilizing effect. There are conformational changes in protein structure after binding of substrate, leading to decrease of the distance between Phe 338 and Tyr 124 as proved works with 4-oxo-N,N,N-trimethylpentanaminium (acetylcholine analogue) [22]; thus these residues could become active in interaction with this double bond in bicycloheptenyl structure. Conformational changes are fundamental and would explain uncompetitive mechanism of inhibition. Nevertheless, this interaction is alleged and more investigation should be done prior to this conclusion. Results from experiment are summarized in Table 2.

**Figure 5:**
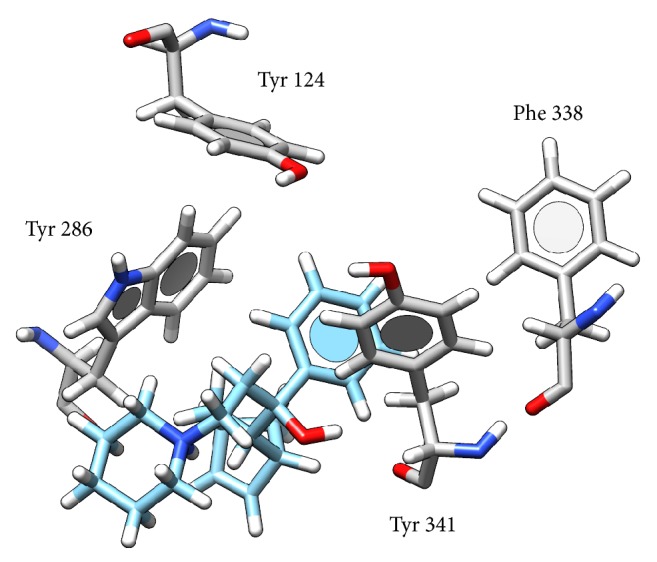
Possible interactions of biperiden with *β*-anionic site of AChE. Atom color: white = H, grey (in AChE) or light blue (in biperiden) = C, blue = N, and red = O.

There is another possible interaction followed with release of free binding energy Δ*G* = −7.58 kcal/mol, where H-bond (2.24 Å) is also predicted between biperiden hydroxyl and O atom in Tyr 341. Additionally, further stabilization seems to be provided by *π*-*π* interaction of benzene ring in biperiden with Trp 286 (3.18 Å), Tyr 341 (3.96 Å), and Phe 338 (4.01 Å) in peripheral anionic subsite and cation-*π* interaction with Tyr 124 (5.84 Å) of AChE (Figure 5).

Biperiden was shown to be weak inhibitor of AChE. For example, most common used cholinesterase's inhibitors like tacrine or donepezil are, respectively, almost 18,500 times and 300,000 times stronger inhibitors of AChE than biperiden [23]. Limited data about biperiden in humans are available. Presumed plasma concentration of biperiden of 5 ng/ml (16 nmol/l) is reached after 1.5 hours when two 2 mg tablets are taken [24]. Typical daily dose is ranging between 2 and 8 mg [25, 26]. However, for causing significant decrease of AChE activity, 342 *μ*g/ml (1.1 mmol/l) is needed as we found out in present work; this means approx. 14 *μ*mol/l for average weight person (80 kg). For humans LD_50_ is not available; for animal models, for example, for rats it is 750 mg/kg (orally); it is approx. 3.0 *μ*mol/l in molar scale. As seen above, for inhibition effect of AChE almost 69,000 times higher dose compared to therapeutic one is required; moreover toxic effect is observed at the same level as needed for causing AChE inhibition. Nevertheless, these results are only hypothetical. Penetration of biperiden to tissues and through blood-brain barrier is based on high lipophilicity and furthermore it is not substrate for efflux transporters like P-glycoprotein which eliminate drugs back into blood flow [27, 28]. At peak concentration of biperiden in plasma, there is approx. 26% occupancy of mAChR in human brain [26]; however no evidence about molar levels in cerebrospinal fluid was found and hence more investigation could be done this way. On the other hand, cerebrospinal fluid levels of, for example, donepezil, were observed [29] and recently changes in concentration between doses were evaluated [30]. Although biperiden is weak inhibitor of AChE and seems to be toxic in high doses, future research based on similar structure derivatives could open interesting direction of AChE inhibitors synthesis and thus in Alzheimer or another neurodegenerative disease treatment.

## 4. Conclusion

Biperiden was revealed to be inhibitor of AChE in our experiment. However, in comparison with standardly used inhibitors, biperiden is weak inhibitor of AChE; *K*_*i*_ was calculated to be 1.11 mmol/l. In real conditions concentration is too low to cause significant inhibition effect; however in high doses it could become toxic. Future perspectives are seen in further investigation of similar derivatives which could create direction in AChE inhibitors research.

## Figures and Tables

**Table 1:** Data from inhibition assay.

Substrate (mM)	Slope (s × l/mol^2^)	Interception (s/mol)	Correlation coefficient
1.0	1.30 × 10^9^	1.60 × 10^6^	0.9647
0.50	1.55 × 10^9^	1.75 × 10^6^	0.9978
0.25	1.87 × 10^9^	2.05 × 10^6^	0.9965

**Table 2:** Found results about biperiden interaction with AChE.

Parameter	Findings
Mechanism of inhibition	Uncompetitive
Inhibition constant (equal to IC_50_ in this kind of inhibition)	1.11 ± 0.20 mmol/l
Predicted binding energy Δ*G*	−7.84 kcal/mol
Predicted interactions	H-bond: Tyr 341 *π*-*π* interaction: Tyr 72, Trp 286, Tyr 341
